# The Impact of Body Weight Changes versus Exercise Capacity Changes on Health-Related Factors following a Lifestyle Intervention in Employees with Metabolic Syndrome

**DOI:** 10.3390/nu14214560

**Published:** 2022-10-29

**Authors:** Pauline Bayerle, Sven Haufe, Momme Kück, Gudrun Protte, Arno Kerling, Simone Ewers, Hedwig Theda Boeck, Thorben Sundermeier, Ralf Ensslen, Kai G. Kahl, Axel Haverich, Uwe Tegtbur, Lars Nachbar

**Affiliations:** 1 Institute of Sports Medicine, Hannover Medical School, 30625 Hannover, Germany; 2 Volkswagen AG, 38440 Wolfsburg, Germany; 3 Department of Psychiatry, Social Psychiatry and Psychotherapy, Hannover Medical School, 30625 Hannover, Germany; 4 Department of Cardiac, Thoracic, Transplantation and Vascular Surgery, Hannover Medical School, 30625 Hannover, Germany

**Keywords:** physical activity, telemonitoring, nutrition, metabolic syndrome, company employees, health-related quality of life

## Abstract

Background: Lifestyle changes are a cornerstone in the treatment of metabolic syndrome (MetS). However, evidence as to which components of the MetS and associated aspects of quality of life are driven by weight loss or improvements in exercise capacity are scarce. Methods: Company employees (*n* = 302, 48.2 ± 8.2 years, BMI 33.2 ± 5.4 kg/m^2^) with diagnosed MetS were evaluated after a 6-month telemonitoring-supported intervention (counselling in nutrition and physical activity) or wait-list control (delayed start of the same intervention). Results: Exercise capacity, body mass index (BMI), and MetS severity were improved after the intervention. Multivariable regression models revealed that changes in BMI were associated with changes in three components of MetS (waist circumference, triglycerides, blood glucose), whereas changes in exercise capacity only were associated to one MetS component change (systolic blood pressure) but also improvements in anxiety severity, aspects of quality of life, and work ability. Conclusions: Both physical activity promotion and diet should be part of a holistic treatment of patients with MetS. However, our data suggest that dietary-induced weight loss might be more successful when aiming at improving MetS risk factors, whereas focusing more on physical activity promotion might be preferred when targeting aspects in quality of life and mental health.

## 1. Introduction

The metabolic syndrome (MetS) is defined as a cluster of three or more of the following five risk factors for cardiovascular disease: abdominal obesity with increased waist circumference, insulin resistance, dyslipidemia, and elevated blood pressure [1]. People with MetS are prone to develop extant cardio-metabolic disease and several forms of cancer [2,3], which induces high healthcare- and socio-economic costs [4,5]. The main non-pharmacological therapeutic approaches for MetS and MetS-related comorbidity prevention and management are increases in physical activity (PA) and dietary modifications inducing weight loss [6,7,8].

Certainly, the prevalence of obesity and physical inactivity is still increasing [9,10]. Both negatively affect cardiovascular, metabolic, and mental health, along with decreases in self-rated aspects of health-related quality of life (HrQoL) [11,12,13,14]. Interventions addressing weight loss or cardio-respiratory fitness (CRF) have shown benefits for several cardio-metabolic biomarkers and HrQoL [11,15]. In clinical practice, most programs combine PA promotion and dietary interventions [16,17]. On average, these programs have shown success for certain outcomes, albeit to different extents [6,18,19,20]. There are some data suggesting that certain health derangements respond stronger to increases in CRF, whereas others respond stronger with losing body weight [8,17]. However, these data are limited and conflicting [21]. Even fewer data on this issue exist for patients with diagnosed MetS.

However, which parameters of the MetS and which MetS-associated psychological aspects, such as depression and anxiety or HrQoL, are driven by dietary changes or PA-related parts of a combined intervention are unknown and the objective of the current analyses.

## 2. Materials and Methods

### 2.1. Study Design and Participants

The present work is a secondary analysis of a previously published study in which the parameters of MetS, physical capacity, work ability, and markers of HrQoL of employees diagnosed with MetS were analyzed after a combined intervention focusing on PA and nutrition [22]. This study was a prospective, randomized, and single-blind (assessor blind) trial conducted as a collaborative project between Volkswagen AG and Hannover Medical School (ClinicalTrials.gov Identifier: NCT03293264). The institutional review board of Hannover Medical School approved the study (No. 7531), and written informed consent was obtained prior to inclusion of study participants.

According to pre-study-defined inclusion and exclusion criteria, we included female and male employees of the main Volkswagen factory in Wolfsburg (Lower Saxony, Germany). Participants had to be over the age of 18, fulfilling at least three of the five MetS components according to the AHA/NHBLI criteria [1], and not participating in an ongoing occupational health program. Exclusion criteria were acute or chronic infections, oncological diseases, joint replacements or any surgery within the last six weeks, pregnant or breast feeding women, and any condition that precluded participation in an exercise intervention.

### 2.2. Study Sample

**Figure 1:**
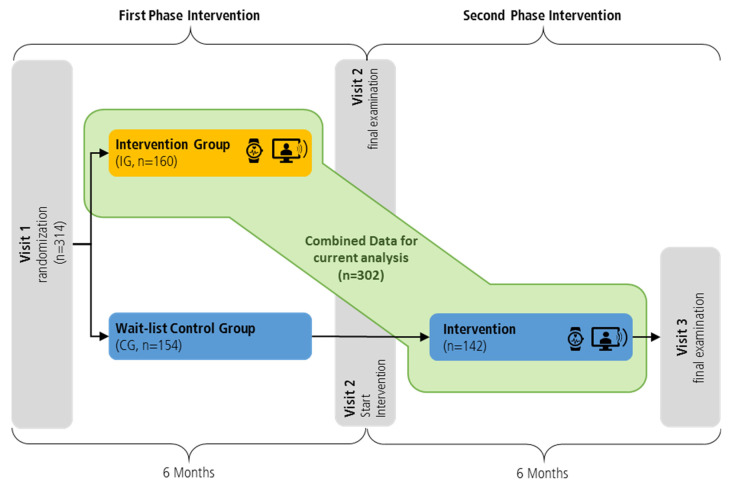
Study design and randomization.

Initially, participants were randomized 1:1 into an intervention group (IG, *n* = 160) and a wait-list control group (CG, *n* = 154) using a computer-based list of random numbers with variable block length to avoid selection bias due to predictability. At baseline (visit 1), the IG started their 6-month telemonitoring-supported exercise intervention, whereas the CG was asked to maintain their usual lifestyle. After 6 months at visit 2, both study groups received the same examinations as conducted at baseline (visit 1). After visit 2, the initial CG was offered to obtain the same 6-month telemonitoring-supported exercise intervention as the IG during the first phase intervention. In summation, both study groups received the same type of intervention and examination, each conducted 6 months apart. (see also Figure 1) Data from the initial IG during the first phase intervention and data from the CG during the second phase intervention were combined for the current analyses.

### 2.3. Intervention

The study intervention was described in detail in the online supplement and elsewhere [22,23]. In brief, during the intervention phase, participants received individualized guidelines for physical training and general recommendations on an active lifestyle. The intervention aimed to increase or keep everyday activity at a high level and to meet the WHO activity target of at least 150 min of moderate endurance training (at least 65% of individual HR_max_) per week [24]. To assist with telemonitoring, motivation, and exercise monitoring, participants were asked to use a provided wearable activity device to be worn on the non-dominant wrist throughout the day (Forerunner 35, Garmin Deutschland GmbH, Garching, Germany).

Prior to each examination, participants completed a 7-day food diary, which was analyzed and reviewed by dietitians for macronutrient and micronutrient content using professional nutrition analysis software (DGE-PC professional Version 5.1.0.048, DGE; Germany). Based on this diary, participants received nutritional counseling according to the recommendations of the German Society for Nutrition [25].

In order to review the initially prescribed training goals and to adjust them if necessary, monthly personal consultations with sports scientists from Hannover Medical School took place on the factory premises. Guidance on nutrition was also individually addressed in the monthly consultations. A specially provided smartphone app (REBIRTH active app, d.velop AG, Gescher) was used as an additional communication medium. The app included information on the further course of the study, exercise opportunities and recommendations, as well as information on a healthy diet.

### 2.4. Examination Instruments

Anthropometric data (body weight, height, waist circumference) were assessed in a standardized way. Fat-free mass and fat mass as markers of body composition were estimated by segmental multi-frequency bioimpedance analysis (InBody720; Biospace, South Korea). Resting blood pressure was measured after 5 min of rest with a suitable automatic blood pressure cuff (Critikon, Dinamap, Boston, MA, USA) as the mean value of two successive recordings.

Exercise capacity (in watt_max_ and watt/kg_bw_) and maximum heart rate (HR_max_) were assessed with a ramp test until subjective exhaustion on a bicycle ergometer (Schiller 911, SCHILLER Medizintechnik GmbH, Feldkirchen b. Munich).

We distributed a questionnaire for the estimation of anxiety severity and depression severity (HADS) [26]. Scores for the anxiety and depression subscales ranged from 0 to 21, with higher scores indicating more severe anxiety or depression [26]. HrQoL was assessed with the short form 36 questionnaire (SF-36) [27]. The SF-36 questionnaire measures HrQoL with eight subscales resulting in two sum scales, the mental and physical component scores. For both scales, a score of 0 points represents a minimum and a score of 100 points a maximum HrQoL. To calculate the total and exercise-related PA as metabolic equivalents of task in (MET)-hours per week the Freiburger Physical Activity questionnaire was applied [28]. To determine work ability, a corresponding questionnaire was used (work ability index [WAI]) [29]. The WAI questionnaire contains seven questions concerning work, work ability, and health, resulting in a total score ranging from 7 to 49, with higher values representing greater work ability.

### 2.5. Statistical Analysis

After combining data from the initial IG with data from the wait-list CG (delayed start of the intervention) into a single intervention dataset, tests for normal distribution were performed using the Kolmogorov–Smirnov test. For non-parametric variables, we compared values with the Mann–Whitney U-Test, and for parametric values with Student’s *t*-tests for unpaired samples. Parametric values were reported as mean and standard deviation (SD); non-parametric values were reported as median and minimum and maximum values. For all outcomes, the analysis was carried out according to the intention-to-treat (ITT) principle, including all randomized subjects. Missing values were replaced by the baseline observation carried-forward method. As a sensitivity analysis, we also conducted a per-protocol analysis including only participants with complete values at baseline and after the intervention. For descriptive analysis, absolute frequencies were calculated for categorical variables and mean and SD for continuous variables. To test for within-group differences from baseline to end of intervention a two-sided Student’s *t*-test for paired samples was used. Bivariate relations between changes in parameters from baseline to 6 months of the intervention were tested using Pearson’s correlation coefficient. To analyze subgroups, we grouped our cohort into tertiles of BMI changes and further subgrouped for high and low changes in absolute exercise capacity. Then, we used a two-sided Student’s *t*-test for unpaired samples to compare changes in parameters of interest between the subgroups of high and low changes in exercise capacity within any tertile of BMI changes. Multivariable linear regression was used to estimate the associated role of intervention-induced changes in BMI and exercise capacity changes with the 6-month intervention in several parameters of interest. As assumption for these analyses, we tested for collinearity and for variance and independence of the residuals. For all analyses, absolute exercise capacity (watt_max_) was used. The type-I-error was set to 5% (two-sided). All statistical analyses were performed with IBM SPSS 27 Statistics (IBM Corporation, New York, NY, USA).

## 3. Results

The combined dataset consisted of 302 participants, from which 248 completed their 6-month intervention period, with 54 subjects dropped out during their intervention period. We documented no serious adverse event during the intervention in both groups. Table 1 shows subject characteristics at baseline for anthropometric and exercise related parameters as well as work ability, anxiety and depression severity, and HrQoL by questionnaire at the start of the intervention.

### 3.1. Physical Activity, Exercise Capacity and Nutritional Intake

Questionnaire-estimated exercise activities increased during the 6-month intervention (pre: 6.67 ± 10.48; post: 13.63 ± 17.51 MET-hours/wk; *p* < 0.001). With the intervention, participants reduced intake of total energy (pre: 2224 ± 1039 kcal; post: 2057 ± 1023 kcal) and total cholesterol (pre: 214 ± 46 mg/dl; post: 207 ± 44 mg/dl) (both *p* = 0.007), whereas the intake of protein (pre: 19.5 ± 3.6%; post: 20.2 ± 3.8%; *p* = 0.008) and omega-3 fatty acid ((% of total fat intake) pre: 0.81 ± 0.33; post: 0.86 ± 0.42; *p* = 0.041) was increased.

Body weight and BMI decreased with the 6-month intervention (−3.4 ± 4.9 kg; −1.06 ± 1.54 kg/m^2^; both *p* < 0.001). The maximum power output during incremental exercise testing was increased after the intervention period (watt_max_ pre: 178.1 ± 37.9; post: 191.3 ± 40.8 watt; *p* < 0.001), whereas the MetS-z-Score was significantly reduced (pre: 0.96 ± 0.68; post: 0.72 ± 0.70; *p* < 0.001). Among the five MetS components, all factors except HDL-cholesterol improved (blood pressure sys/dia (mmHg) pre: 137/89 ± 13/9; post: 133/86 ± 13/9; *p* < 0.001; waist circumference (cm) pre: 114.7 ± 12.6; post: 110.70 ± 13.2; *p* < 0.001; triglycerides (mg/dL) pre: 196.0 ± 157.4; post: 170.1 ± 134.0; *p* < 0.001; fasting plasma glucose (mg/dL) pre: 110.6 ± 24.4.; post: 106.5 ± 22.1; *p* < 0.001; HDL cholesterol (mg/dL) pre: 44.2 ± 9.6; post: 44.5 ± 9.2; *p* = 0.171).

### 3.2. Association of Health- and Work-Related Outcomes

**Figure 2:**
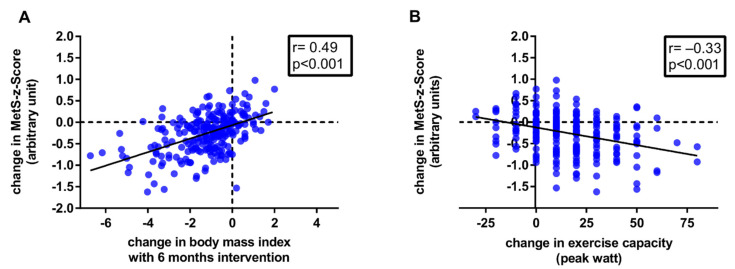
6-month intervention induced changes in MetS severity (MetS-z-Score) in correlation with changes in BMI (**A**) and changes in absolute exercise capacity (**B**).

After 6 months intervention, changes in BMI were significantly correlated to changes in the HADS depression score (r = 0.19, *p* = 0.001), the physical score of SF-36 (r = −0.24, *p* < 0.001), WAI (r = −0.16, *p* = 0.005), and to changes in features of MetS and the MetS-z-Score (see Table 2 and Figure 2A) but not to changes in the HADS anxiety score (r = 0.09, *p* = 0.121) or mental score of SF-36 (r = −0.11, *p* = 0.070).

Changes in exercise capacity (watt_max_) were correlated to changes in the HADS depression score (r = −0.22, *p* < 0.001), the HADS anxiety score (r = −0.16, *p* = 0.010), mental score of SF-36 (r = 0.21, *p* < 0.001), physical score of SF-36 (r = 0.15, *p* = 0.019), WAI (r = 0.20, *p* < 0.001), and to changes in features of MetS except for HDL-cholesterol, and the MetS-z-Score (see Table 2 and Figure 2B, and Table S1 for the per protocol analysis).

In multivariate linear regression models including age, sex, changes in absolute exercise capacity, changes in BMI, and the respective baseline values of the dependent variable, it turned out that changes in BMI were associated with changes in waist circumference, triglycerides, blood glucose, and the physical score of SF-36, whereas changes in exercise capacity were associated with changes in systolic blood pressure, anxiety severity, the mental score of SF-36, and work ability (for details see Table 3), and Table S2 for the per protocol analysis.

**Figure 3:**
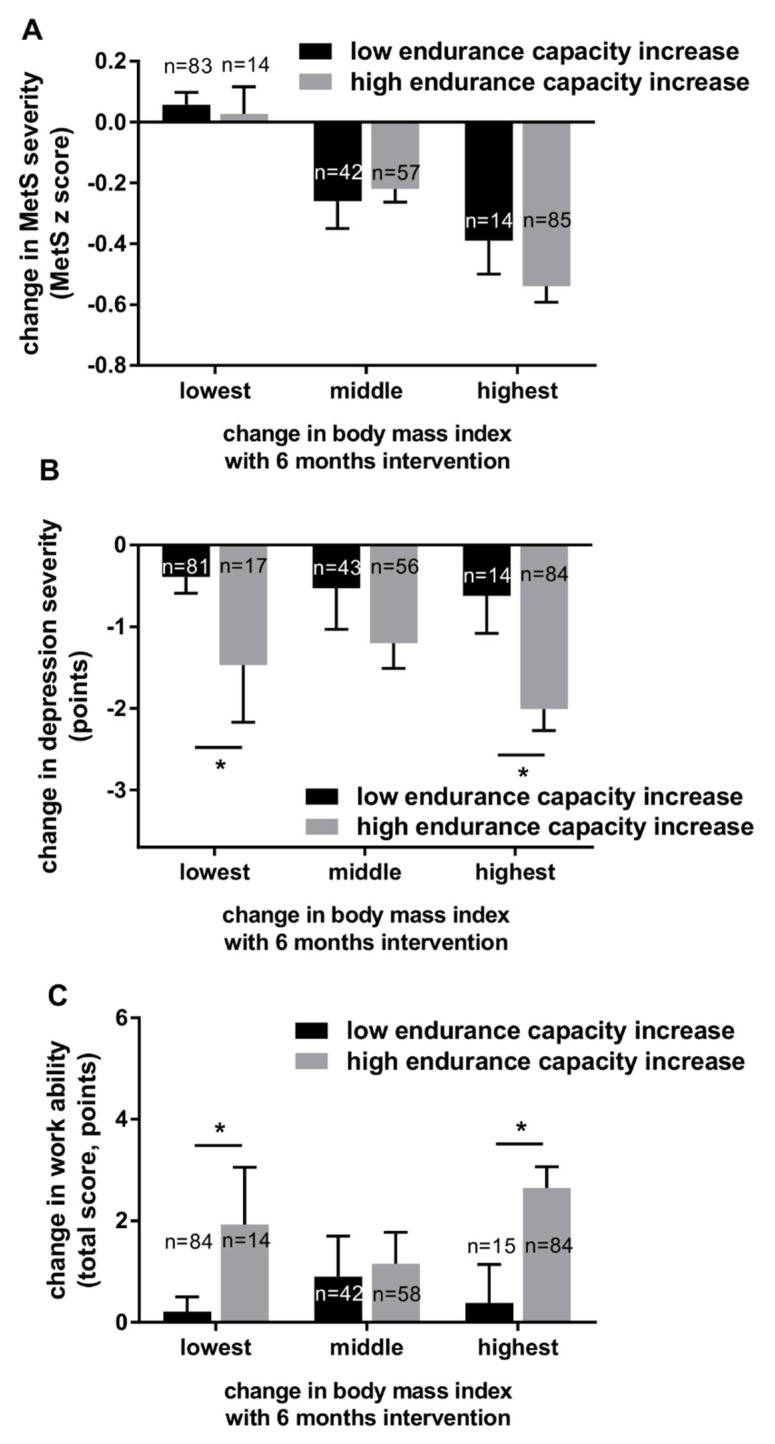
6-month intervention induced changes in MetS severity (**A**), changes in depression severity (**B**), and changes in work ability (**C**) classified into tertiles of BMI changes and further subgrouped for changes in exercise capacity (low: black columns; high: grey columns) during the intervention. Data are mean ± SEM, * *p* < 0.05, significantly different for absolute exercise capacity changes among the same BMI change tertile.

To further shed light on the independent influence of changes in exercise capacity versus changes in BMI we present changes in MetS severity, depression severity, and work ability classified into changes in BMI during the intervention (tertiles) and then further subdivided into groups with high versus low changes in exercise capacity during the 6-month intervention (Figure 3). For changes in MetS severity, exercise capacity changes had no significant influence in addition to the influence of changes in BMI (see Figure 3A). In contrast, for changes in depression severity and work ability, the degree of changes in exercise capacity had a separate impact on these outcomes in addition to the influence of changes in BMI (see Figure 3B,C).

## 4. Discussion

Conservative treatment of MetS and associated comorbidities typically focus on a combined strategy including a healthy lifestyle, appropriate diet and nutritional habits, as well as on sufficient PA [8]. However, to treat a health-related condition more individually, it would be helpful to shed more light on what disease-related markers respond stronger to the PA part or to the dietary part of an intervention.

The worldwide trends in insufficient PA, on the one hand, and increasing obesity rates, on the other hand, are known risk factors for overall public health [9,10,30] and for the development of the MetS [31,32]. Obesity is a main component of the MetS, and is closely linked to the other four components [30,31]. Therefore, weight reduction is a primary goal of the treatment and serves as a preventive measure [32]. However, in practice, weight loss is difficult to achieve and even harder to maintain [33]. Notably, improvement in MetS severity is also possible by increases in activity-induced exercise capacity, even in the absence of body weight loss [34]. In this regard, the obesity paradox was described in several studies, suggesting that fitness markedly alters the relationship between adiposity and prognosis in chronic diseases, particularly in cardiovascular disease [32,35]. Accordingly, high levels of CRF are likely to improve prognosis and all-cause mortality [32].

Our analyses show that both changes in BMI and changes in exercise capacity after a combined intervention were correlated to changes in the five components of the MetS. This is in line with studies showing that single interventions focusing on diet or PA are successful for the treatment of MetS [34,36,37]. We also observed that changes in associated “comorbidities” of MetS such as HrQoL, work ability, or anxiety and depression severity are responsive to both treatment components. However, because of the strong interrelation between changes in weight and changes in CRF in a combined intervention, it is likely that some of our observed univariate relationships are biased by changes in the other part of the intervention.

To address the shortcomings of univariate testing, we conducted multivariate analyses analyzing independent effects of weight loss changes and CRF changes on outcomes of interest. These analyses implicate that weight loss is a stronger influence for physiological features of MetS but has lesser impact on mental and work-related features when compared to changes in exercise capacity. This suggests that dietary induced weight loss is of stronger impact when addressing cardiovascular risk factors such as waist circumference, blood lipids, or blood glucose concentration, which was reported before [17,38]. In this regard, the Mediterranean Diet, in particular, showed beneficial effects on disease-related interventions with a focus on nutrition, with cardiovascular disease and MetS being the most investigated diseases for Mediterranean Diet [39]. The beneficial effects could be primarily related to its anti-inflammatory and anti-oxidant properties, as well as the effectiveness of this dietary pattern in controlling waist circumference and obesity [39].

On the other hand, our results strengthen previous studies showing that PA is suited to improve mental “comorbidities” of MetS [40,41,42]. In particular, mental and work-related parameters (anxiety severity, mental component of HrQoL, work ability) responded stronger and independent from changes in BMI to changes in exercise capacity. Our results may have relevance for MetS management. For example, if individuals have higher grades of obesity or worsened blood parameters, body weight loss should be the main focus of the intervention. On the other hand, for a person with lower MetS severity but impairments in the mental components of health or coping with working demands, a tailored PA program seems an important tool for an optimized treatment.

### Strengths and Limitations

Our study has strengths and limitations. It was not a pre-specified approach to evaluate both the initial intervention group and the delayed intervention period of the initial wait-list control as a whole. However, we did so in favor of greater statistical power. Notably, there were certain differences in baseline values of relevant parameters (before the start of the 6-month intervention) between the combined study groups.

Many unknown factors in addition to the supervised physical activities might have influenced the measured outcomes, including stress management, smoking behavior, or selection of participants. There is evidence that social status and workplace characteristics play a role, with links between a lower social status and an increased disease risk. Our cohort was recruited from one company with participants who were very well informed about the disease and highly motivated to actively improve their health status through lifestyle changes. Furthermore, a series of information events were held on the factory premises. These points could have contributed to the overall positive outcomes of the study and limited the generalizability of the obtained study results. In addition, despite certain statistical tests, we cannot rule out the possibility that the change in BMI and change in exercise are interrelated and the association between changes in other parameters might be a spurious association. Finally, we cannot estimate long-term effects on changes in MetS severity in our participants because of the relatively short duration of our intervention.

## 5. Conclusions

In general, both activity and dietary changes should be addressed in patients with MetS, as data suggest that particularly the combination of diet and physical activity reduces disease risk factors [17]. Exercise in addition to dietary-induced weight loss is also crucial to preserve muscle mass as metabolically important tissue. However, our data might help to more individually set the applied proportion of these two treatments parts for an optimized and more tailored outcome on MetS components and associated derangements in markers of QoL, mental health, and work ability.

## Figures and Tables

**Table 1:** Subject Characteristics at Start of Intervention.

	Total Group (*n* = 302)		Intervention Group (*n* = 160)		Delayed Intervention Group (Former Wait-List Control) (*n* = 142)		
		*n*		*n*		*n*	*p*-Value
Age (years)	50 [24; 63]	302	50 [25; 62]	160	50 [24; 63]	142	0.911
Body weight (kg)	105.0 [62.2; 165.1]	302	106.5 [67.6; 158.0]	160	102.1 [62.2; 165.1]	142	0.140
Waist circumference (cm)	113.8 [89.0; 153.5]	299	115.0 [92.0; 153.5]	158	112.0 [89.0; 151.0]	141	0.145
Body mass index [BMI(kg/m^2^)]	32.5 [22.9; 49.5]	302	32.9 [22.9; 49.3]	160	31.9 [23.5; 49.5]	142	0.075
Body fat (%)	32.3 [14.4; 56.5]	297	32.5 [14.4; 53.4]	158	32.2 [16.1; 56.5]	139	0.331
Fat Free Mass (kg)	71.3 ± 11.7	297	71.2 ± 11.1	158	71.4 ± 12.5	139	0.875
Systolic BP (mmHg)	135.8 [106.0; 181.0]	298	136.0 [108.0; 181.0]	157	133.0 [106.0; 170.0]	141	0.010
Diastolic BP (mmHg)	88.8 [67.0; 120.0]	298	88.5 [67.5; 113.0]	157	89.0 [67.0; 120.0]	141	0.186
HbA1c (%)	5.4 [4.6; 12.0]	295	5.4 [4.7; 12.0]	154	5.3 [4.6; 9.0]	141	0.016
Total cholesterol (mg/dL)	214.3 ± 46.2	302	215.3 ± 45.9	160	213.0 ± 46.7	142	0.670
HDL cholesterol (mg/dL)	42.9 [21.5; 78.5]	301	43.7 [25.6; 78.5]	159	42.4 [21.5; 71.2]	142	0.287
LDL cholesterol (mg/dL)	136.2 ± 38.9	302	137.8 ± 38.8	160	134.5 ± 39.2	142	0.462
MetS-z-Score	0.89 [−0.45; 4.13]	287	0.91 [−0.45; 4.13]	157	0.87 [−0.34; 3.16]	130	0.544
Every day activity (MET-h/wk)	16.4 [0.0; 140.1]	267	14.3 [0.0; 129.7]	150	24.4 [0.4; 140.1]	117	<0.001
Sports related activity (MET-h/wk)	1.5 [0.0; 84.0]	267	0.0 [0.0; 84.0]	150	4.5 [0.0; 56.0]	117	0.001
Total physical activity (MET-h/wk)	23.2 [0.0; 140.1]	267	18.6 [0.0; 129.7]	150	30.8 [1.2; 140.1]	117	<0.001
Relative exercise capacity (watt/kg)	1.71 ± 0.42	301	1.66 ± 0.40	160	1.78 ± 0.44	141	0.018
Exercise capacity (watt_max_)	180.0 [80.0; 320.0]	301	180.0 [80.0; 320.0]	160	190.0 [80.0; 280.0]	141	0.064
Work ability (WAI)	38.0 [16.0; 49.0]	301	38.0 [21.0; 48.0]	159	39.0 [16.0; 49.0]	142	0.026
HADS_subscale anxiety	5.0 [0.0; 16.0]	289	5.0 [0.0; 15.0]	159	4.0 [0.0; 16.0]	130	0.001
HADS_subscale depression	3.0 [0.0; 15.0]	289	3.0 [0.0; 15.0]	159	3.0 [0.0; 15.0]	130	0.465
SF-36_physical score	50.8 [23.3; 64.5]	283	49.8 [24.5; 64.5]	155	51.9 [23.3; 61.3]	128	0.047
SF-36_mental score	52.9 [16.5; 66.1]	283	51.9 [16.5; 66.1]	155	54.0 [20.9; 63.7]	128	0.022

Parametric values were reported as mean and standard deviation, non-parametric values were reported as median and min and max values. BP = blood pressure; HDL = high density lipoprotein; LDL = low density lipoprotein; MET = metabolic equivalent of task.

**Table 2:** Correlation of Intervention–induced Changes in BMI and Exercise Capacity with Changes in Features of MetS.

	Delta Waist Circumference	Delta Triglycerides	Delta HDL chol.	Delta Glucose conc.	Delta Systolic BP
delta BMI (kg/m^2^)	0.73 *	0.32 *	−0.17 *	0.27 *	0.25 *
delta exercice capacity (watt_max_)	−0.32*	−0.24 *	0.07	−0.20 *	−0.24 *

Univariate correlation between changes in BMI and changes in absolute exercise capacity during the 6-month intervention, respectively, with changes in the five components of the metabolic syndrome were analyzed. Pearson’s correlation coefficients and significance level are shown (* indicates *p* < 0.01).

**Table 3:** Multivariable Regression Analyses between 6–month Intervention-induced Changes in MetS Components and Components of HrQoL.

	**Dependent Variable**									
	**Delta Waist Circumference**		**Delta Triglycerides**		**HDL Cholesterol**		**Delta Blood Glucose**		**Delta Systolic BP**	
**Independent Variables**	**Coefficient** **β**	***p*** **-Value**	**Coefficient** **β**	***p*** **-Value**	**Coefficient** **β**	***p*** **-Value**	**Coefficient** **β**	***p*** **-Value**	**Coefficient** **β**	***p*** **-Value**
delta BMI	**0.71**	<0.001	**0.27**	<0.001	−0.15	0.061	**0.23**	0.003	0.10	0.085
delta exercise capacity	−0.03	0.543	−0.07	0.281	0.01	0.772	−0.05	0.386	**−0.14**	0.038
age	−0.02	0.721	0.02	0.668	0.09	0.088	0.04	0.409	−0.05	0.282
sex	−0.01	0.738	0.02	0.754	**−0.19**	0.009	0.05	0.351	0.10	0.074
baseline value of the respective	0.01	0.881	**−0.51**	<0.001	**−0.40**	<0.001	**−0.48**	<0.001	**−0.46**	<0.001
	**Dependent Variable**									
	**Delta HADS Anxiety**		**Delta HADS Depression**		**Delta Physical Score SF** **–** **36**		**Delta Mental Score SF–36**		**Delta WAI Total Score**	
**Independent Variables**	**Coefficient** **β**	***p*** **-Value**	**Coefficient** **β**	***p*** **-Value**	**Coefficient** **β**	***p*** **-Value**	**Coefficient** **β**	***p*** **-Value**	**Coefficient** **β**	***p*** **-Value**
delta BMI	0.06	0.312	0.05	0.264	**−0.19**	0.009	0.04	0.488	−0.07	0.218
delta exercise capacity	**−0.15**	0.042	−0.12	0.051	0.10	0.218	**0.17**	0.010	**0.19**	0.002
age	−0.02	0.718	0.01	0.823	−0.01	0.941	0.04	0.416	−0.05	0.345
sex	0.07	0.201	−0.01	0.839	0.06	0.309	0.01	0.802	0.06	0.271
baseline value of the respective	**−0.38**	<0.001	**−0.54**	<0.001	**−0.44**	<0.001	**−0.44**	<0.001	**−0.41**	<0.001

Multivariable linear regression analyses with different dependent variables of interest (e.g., delta waist circumference). Independent variables for any shown analysis were delta BMI, delta absolute exercise capacity, age, sex, and the baseline value of the respective independent variable (e.g., baseline value of waist circumference) as listed in the first row. For any dependent variable, the association with the independent variables are given as the standardized coefficent beta (β) and the respective *p*-value.

## Data Availability

The datasets used and/or analyzed during the current study are available from the corresponding author on reasonable request.

## Supplementary Materials

The following supporting information can be downloaded at: https://www.mdpi.com/article/10.3390/nu14214560/s1, Supplementary Text and Tables S1 and S2 for a per protocol analysis of our data in addition to the ITT analysis shown in the article. Tables S1: Univariate correlation between changes in BMI and changes in exercise capacity during the 6-month intervention, respectively, with changes in the five components of the metabolic syndrome were analyzed. Pearson´s correlation coefficients and significance level are shown (* indicates *p* > 0.01); Table S2: Multivariable linear regression analyses with different dependent variables of interest (e.g., delta waist circumference). Independent variables for any shown analysis were delta BMI, delta absolute exercice capacity, age, sex, and the baseline value of the respective independent variable (e.g., baseline value of waist circumference) as listed in the first row. For any dependent variable the association with the independent variables are given as the standardized coefficent beta (β) and the respective *p*-value.title.
