# Supplementary material for: The Impact of Body Weight Changes versus Exercise Capacity Changes on Health-Related Factors following a Lifestyle Intervention in Employees with Metabolic Syndrome
Source: Nutrients. 2022 Oct 29;14(21):4560. doi: 10.3390/nu14214560 (PMC9655595; doi:10.3390/nu14214560)
Supplement: Supplementary file 1 [file nutrients-14-04560-s001.zip › nutrients-1966133-supplementary.pdf]

SUPPLEMENTAL DATA

In addition to the intention to treat analyses (ITT) in the main body of the manuscript we here show results of a per protocol analyses. In this analysis, only participants with complete values at baseline and after the intervention (n=248) are included in the calculations. Otherwise, the calculations are conducted as described in the statistical section of the article.

Correlation analyses for changes in BMI and changes in exercise capacity

After 6 months intervention, changes in BMI were significantly correlated to changes in the HADS depression score (r= 0.14, p=0.028), the physical score of SF–36 (r= –0.24, p<0.001), the WAI (r= –0.13, p=0.046) and to changes in features of MetS (see table S1) and the MetS–z–Score (r= 0.47, p<0.001) but not to changes in the HADS anxiety score (r= 0.09, p=0.121) or mental score of SF–36 (r= –0.11, p=0.070).

Changes in exercise capacity ($watt_{max}$) were correlated to changes in the HADS depression score (r= –0.18, p=0.005), the mental score of SF–36 (r= 0.18, p=0.005), WAI (r= 0.17, p=0.007), and to changes in feature of MetS except for HDL–cholesterol (see table S1), and the MetS–z–Score (–0.22, p<0.001), but not to changes in the HADS anxiety score (r= –0.11, p=0.090), the physical score of SF–36 (r= 0.10, p=0.134).

Table S1. Correlation of intervention–induced changes in BMI and exercise capacity with changes in features of MetS

	delta waist circum-ference	delta tri-glycerides	delta HDL chol.	delta glucose conc.	delta systolic BP
**delta BMI (kg/m^2)**	0.69*	0.29*	– 0.17*	0.25*	0.21*
**delta exercice capacity (watt)**	– 0.25*	– 0.18*	0.04	– 0.17*	– 0.20*

Univariate correlation between changes in BMI and changes in exercise capacity during the 6-month intervention, respectively, with changes in the five components of the metabolic syndrome were analyzed. Pearson´s correlation coefficients and significance level are shown (* indicates p>0.01)

In multivariate linear regression models including age, gender, changes in absolute exercise capacity, changes in BMI and the respective baseline values of the dependent variable, it turned out that changes in BMI were associated with changes in waist circumference, triglycerides, blood glucose, and the physical score of SF–36, whereas changes in exercise capacity were associated with changes in anxiety severity, the mental score of SF–36 and work ability (for details see supplemental Table S2).

Table S2. Multivariable regression analyses between 6–month intervention induced changes in MetS components and components of HrQoL.

	**dependent variable**									
	**delta waist circumference**		**delta triglycerides**		**HDL cholesterol**		**delta blood glucose**		**delta systolic BP**	
**independent variables**	coefficient β	p-value	coefficient β	p-value	coefficient β	p-value	coefficient β	p-value	coefficient β	p-value
delta BMI	**0.69**	<0.01	**0.25**	<0.01	–0.14	0.02	**0.20**	<0.01	0.10	0,08
delta exercise capacity	0.00	0.99	–0.05	0.40	–0.01	0.88	–0.03	0.64	–0.09	0.12
age	–0.02	0.62	0.03	0.61	0.09	0.11	0.05	0.37	–0.04	0.41
sex	–0.01	0.82	0.01	0.81	**–0.22**	<0.01	0.06	0.28	0.10	0.06
baseline value of the respective	–0.02	0.70	**–0.55**	<0.01	**–0.45**	<0.01	**–0.52**	<0.01	**–0.52**	<0.01
	**dependent variable**									
	**delta HADS anxiety**		**delta HADS depression**		**delta physical score SF–36**		**delta mental score SF–36**		**delta WAI total score**	
**independent variables**	coefficient β	p-value	coefficient β	p-value	coefficient β	p-value	coefficient β	p-value	coefficient β	p-value
delta BMI	0.06	0.31	0.05	0.34	**–0.19**	0.00	–0.02	0.75	–0.04	0.49
delta exercise capacity	**–0.15**	0.04	–0.09	0.09	0.01	0.84	**0.15**	0.04	**0.12**	0.04
age	–0.02	0.71	0.01	0.83	0.01	0.90	0.04	0.44	–0.06	0.30
sex	0.07	0.20	–0.02	0.76	0.08	0.18	–0.02	0.78	0.05	0.39
baseline value of the respective	**–0.38**	**<0.01**	**–0.57**	<0.01	**–0.46**	<0.01	**–0.51**	<0.01	**–0.46**	<0.01

Multivariable linear regression analyses with different dependent variables of interest (e.g. delta waist circumference). Independent variables for any shown analysis were delta BMI, delta absolute exercice capacity, age, sex, and the baseline value of the respective independent variable (e.g. baseline value of waist circumference) as listed in the first row. For any dependent variable the association with the independent variables are given as the standardized coefficent beta (β) and the respective p-value.
